# Increased nature relatedness and decreased authoritarian political views after psilocybin for treatment-resistant depression

**DOI:** 10.1177/0269881117748902

**Published:** 2018-01-17

**Authors:** Taylor Lyons, Robin L Carhart-Harris

**Affiliations:** Psychedelic Research Group, Department of Medicine, Imperial College London, UK

**Keywords:** Psilocybin, nature relatedness, authoritarianism, depression, political perspective

## Abstract

**Rationale:**

Previous research suggests that classical psychedelic compounds can induce lasting changes in personality traits, attitudes and beliefs in both healthy subjects and patient populations.

**Aim:**

Here we sought to investigate the effects of psilocybin on nature relatedness and libertarian–authoritarian political perspective in patients with treatment-resistant depression (TRD).

**Methods:**

This open-label pilot study with a mixed-model design studied the effects of psilocybin on measures of nature relatedness and libertarian–authoritarian political perspective in patients with moderate to severe TRD (*n*=7) versus age-matched non-treated healthy control subjects (*n*=7). Psilocybin was administered in two oral dosing sessions (10 mg and 25 mg) 1 week apart. Main outcome measures were collected 1 week and 7–12 months after the second dosing session. Nature relatedness and libertarian–authoritarian political perspective were assessed using the Nature Relatedness Scale (NR-6) and Political Perspective Questionnaire (PPQ-5), respectively.

**Results:**

Nature relatedness significantly increased (*t*(6)=−4.242, *p*=0.003) and authoritarianism significantly decreased (*t*(6)=2.120, *p*=0.039) for the patients 1 week after the dosing sessions. At 7–12 months post-dosing, nature relatedness remained significantly increased (*t*(5)=−2.707, *p*=0.021) and authoritarianism remained decreased at trend level (*t*(5)=−1.811, *p*=0.065). No differences were found on either measure for the non-treated healthy control subjects.

**Conclusions:**

This pilot study suggests that psilocybin with psychological support might produce lasting changes in attitudes and beliefs. Although it would be premature to infer causality from this small study, the possibility of drug-induced changes in belief systems seems sufficiently intriguing and timely to deserve further investigation.

## Introduction

A growing body of evidence suggests that classical psychedelic compounds such as lysergic acid diethylamide (LSD) (Carhart-Harris et al., 2016b; Schmid and Liechti, 2017), psilocybin (Carhart-Harris et al., 2016a; Griffiths et al., 2016; Ross et al., 2016) and N,N-dimethyltryptamine (DMT) in ayahuasca (Osorio Fde et al., 2015) can promote enduring changes in personality traits, attitudes and beliefs – and similar effects have recently been reported for 3,4-methylenedioxy-methamphetamine (MDMA), which shares some properties with classical psychedelic compounds (Wagner et al., 2017) albeit with some important differences (Carhart-Harris and Nutt, 2017). Focusing more specifically on psilocybin, a single high dose of this naturally occurring compound was associated with enduring increases in trait openness, psychological wellbeing and life satisfaction in healthy volunteers 14 months later (Griffiths et al., 2008; MacLean et al., 2011). Psilocybin has also been shown to improve symptoms of addiction (Bogenschutz et al., 2015; Johnson et al., 2014), anxiety (Griffiths et al., 2016; Grob et al., 2011; Moreno et al., 2006; Ross et al., 2016) and depression (Carhart-Harris et al., 2016a; Griffiths et al., 2016; Ross et al., 2016) when administered in a psychologically supportive setting.

In the general population, psychedelic drug use is not associated with increased incidence of mental health problems (Johansen and Krebs, 2015; Krebs and Johansen, 2013), but is instead associated with lower rates of suicidality and psychological distress (Hendricks et al., 2015a, 2015b; Johansen and Krebs, 2015; Krebs and Johansen, 2013). Psychedelic drug users have also been shown to exhibit greater optimism (or reduced pessimism) than non-users (Grob et al., 1996) as well as increased concern for others, nature and the environment when compared with users of cannabis, amphetamine or heroin (Lerner and Lyvers, 2006). Experience with psychedelics has been found to positively affect one’s sense of feeling part of nature rather than separate from it, leading to pro-environmental behavioural changes (Forstmann and Sagioglou, 2017). Nature relatedness, defined as the subjective sense of connection with the natural environment, is associated with lower levels of anxiety (Capaldi et al., 2014; Martyn and Brymer, 2014), and has been shown to promote psychological wellbeing at both the trait (Cervinka et al., 2012; Howell et al., 2011; Mayer and Frantz, 2004; Nisbet et al., 2011) and state (Mayer et al., 2008; Nisbet and Zelenski, 2011) level. Furthermore, interacting with the natural environment has been shown to improve mood and cognitive functioning in patients with major depressive disorder (MDD) (Berman et al., 2012), and visits to outdoor green spaces of 30 min or more per week has been predicted to reduce the population prevalence of depression by up to 7% (Shanahan et al., 2016). Taken together, these findings indicate that psychedelics can promote enduring changes in personality traits, attitudes and beliefs.

A recent correlational study of ours found that lifetime psychedelic drug use in the general population positively predicted nature relatedness and negatively predicted authoritarian political views, in a manner that appeared to be mediated by acute and temporary ‘ego-dissolution’ (Nour et al., 2017). Moreover, a recent pilot study of ours found rapid and enduring reductions in depressive symptoms in a treatment-resistant depression (TRD) sample after psychologically supported psilocybin therapy (Carhart-Harris et al., 2018a). As part of this project, the present research sought to address potential changes in nature relatedness and political perspective in this sample of TRD patients before versus after psilocybin treatment. Measures were collected at screening, 1 week and 7–12 months post-dosing. To demonstrate robustness to order confounds, we also collected data from an age-matched but non-treated healthy control group, tested over an equivalent period.

## Methods

### Ethical approvals

This study received a favourable opinion from NRES London-West London, was sponsored by Imperial College London, and was carried out in accordance with Good Clinical Practice Guidelines. The National Institute for Health Research/Wellcome Trust Imperial Clinical Research Facility (ICRF) provided site-specific approval and the Medicines and Healthcare products Regulatory Agency (MHRA) reviewed and approved this research. All patients provided written informed consent.

### Study design and participants

This open-label pilot study with a mixed-model design compared the effects of psilocybin on nature relatedness and libertarian–authoritarian political perspective in patients with TRD versus healthy control subjects. The findings presented here are part of a larger open-label pilot study of psilocybin in TRD, the results of which have been reported elsewhere (Carhart-Harris et al., 2016a, 2017). The study participants (*n*=14) and research team were not masked to treatment assignment. All TRD patients (*n*=7) were administered psilocybin in two dosing sessions: an initial safety dose (10 mg) and a subsequent treatment dose (25 mg) 1 week later. The control subjects (*n*=7) were not administered psilocybin and were primarily included to examine test–retest data on the relevant primary measures.

General practitioners were provided with recruitment information via the North West London Clinical Research Network and were asked to identify potential patients with TRD. TRD patients residing in the UK were also able to self-refer to the study. In most cases, it was the TRD patients that initiated contact with the research team. After initiating contact, patients were given a study information sheet and were initially screened via a telephone call with the research team’s lead psychiatrist to determine eligibility for the study. Each patient’s general practitioner or psychiatrist provided written confirmation and documentation of the patient’s diagnosis and mental health background.

The inclusion criteria were (a) MDD of a moderate to severe degree (16+ on the 21-item Hamilton Depression Rating scale [HAM-D]), and (b) no improvement despite two adequate courses of antidepressant treatment of different pharmacological classes lasting at least six weeks within the current depressive episode. The exclusion criteria were (a) current or previously diagnosed psychotic disorder, (b) first-degree relative with a diagnosed psychotic disorder, (c) medically significant condition rendering unsuitability for the study, (d) history of serious suicide attempts (requiring hospitalisation), (e) previous manic episode, (f) blood or needle phobia, (g) positive pregnancy test at screening or during the study, and (h) current drug or alcohol dependence.

Physically and mentally healthy age-matched control subjects were recruited via word of mouth and were subject to the same exclusion criteria outlined above. Informed consent was obtained from all of the study participants, including the controls.

### Drug

Psilocybin was obtained from THC Pharm GmbH (Frankfurt, Germany) and formulated by Guy’s and St Thomas’ Hospitals’ Pharmacy Manufacturing Unit (London, UK). Home Office approvals for storing and dispensing Schedule One drugs were obtained.

### Procedures and dosing sessions

Screening procedures typically lasted 4 h and consisted of psychiatric and physical health assessments. All patients provided written informed consent. Clinicians carried out a Mini-International Neuropsychiatric Interview (Sheehan et al., 1998) and assessed depression severity using the HAM-D (Hamilton, 1960) and Montgomery–Åsberg Depression Rating Scale (MADRS) (Montgomery and Asberg, 1979) in patient participants. Physical assessments included an electrocardiogram, routine blood tests, blood pressure and heart rate tests, and a physical examination. Eligible TRD patients were allocated two psychiatrists for support throughout the study.

All patients enrolled in the study attended a pre-dosing preparatory session with the allocated psychiatrists on the next visit. Psychological preparation consisted of discussing the effects of psilocybin and simulating aspects of the dosing session to make patients aware of what to expect. Patients also shared their personal history and thoughts on the origins of their depression. This session helped to build rapport and trust between the patient and their allocated psychiatrists, thus minimising the risk of adverse reactions to psilocybin (Metzner et al., 1965).

All patients attended two dosing sessions separated by 1 week at the ICRF. A close friend or relative accompanied each patient to and from the ICRF and return transport was arranged before the dosing sessions took place. A urine sample for drugs of abuse (and pregnancy where applicable), breathalyser test for alcohol use and vital signs measurements were collected before each dosing session. Patients were then taken to a decorated dosing room with dim lighting and music where they were invited to lie down and relax. The allocated psychiatrists remained at either side of the bed and adopted a non-directive, supportive approach. Patients were supervised at all times and the psychiatrists checked how they were feeling at approximately 1 h intervals, and assessed them for discharge approximately 6 h after drug administration.

### Outcome measures

To assess political views on the dimension of libertarianism to authoritarianism, a recently validated (Nour et al., 2017) subset of questions (five items) from the Libertarian-Authoritarian Questionnaire (Evans et al., 1996) were used as a short version and termed the Political Perspective Questionnaire (PPQ-5) (Nour et al., 2017). The validated 6-item Nature Relatedness Scale (NR-6) (Nisbet and Zelenski, 2013) was used to measure the subjective sense of connectedness to nature. Self-rated depressive symptoms were measured at baseline and two follow-up time points (1 week and 7–12 months) in all study participants using the Quick Inventory of Depressive Symptoms (QIDS) (Rush et al., 2003). The above measures were assessed at baseline and then again at the 1 week and 7–12-months follow-ups for all study participants.

### Statistical analysis

All statistical analyses were performed using SPSS version 23.0 (IBM Corp., Armonk, NY, USA). Within-group comparisons were performed using one-tailed paired *t*-tests for parametric data and Wilcoxon signed ranks tests for non-parametric data. Between-group comparisons were performed using two-tailed independent *t*-tests for parametric data and Mann–Whitney *U* tests for non-parametric data. We provide 95% CIs around the mean differences. Effect sizes were calculated using the Hedges’ *g* formula due to the small sample size in this study.

## Results

No serious adverse events occurred as a result of the psilocybin administration, and the acute drug effects were well tolerated by all patients (see Carhart-Harris et al. (2016a, 2017a) for more information).

### Demographics

Of the total 14 participants who took part in this study, the majority were Caucasian (78.6%) and men (64.3%) with a post-secondary level of education (85.7%). In the patient sample (*n*=7), baseline HAM-D scores ranged from 24 to 36 (*M*=28.6, *SD*=3.7) and the MADRS scores from 28 to 40 (*M*=35.9, *SD*=5); thus, all patients recruited were diagnosed with TRD of at least moderate severity, with most meeting criteria for severe depression. Control subjects were not clinically assessed because they were deemed physically and mentally healthy. Self-reported depressive symptoms (QIDS) were collected for all study participants. Table 1 outlines participant demographic details and depressive scores at baseline.

**Table 1.** Study participant demographics and baseline depression scores.

Characteristic	Control subjects (*n*=7)	TRD patients (*n*=7)	All participants (*N*=14)	*p*-value
Gender (% male)	29%	100%	64.3%	0.005^a^
Age in years (mean, SEM)	43.3 (6.8)	48.3 (4.5)	45.8 (3.9)	0.550^b^
Ethnicity (%)				0.213^a^
White	71.4%	85.7%	78.6%	
Black	0%	14.3%	7.1%	
Asian	28.6%	0%	14.3%	
Education (%)				1.000^a^
Secondary school	14.3%	14.3%	14.3%	
Undergraduate	57.1%	57.1%	57.1%	
Postgraduate	28.6%	28.6%	28.6%	
Baseline scores (mean, SEM)				
QIDS	3.86 (0.91)	19.43 (1.52)	NA	0.002^c^

a Chi-square test.

b *t* test.

c *U* test.

### Nature relatedness

**Figure 1.**
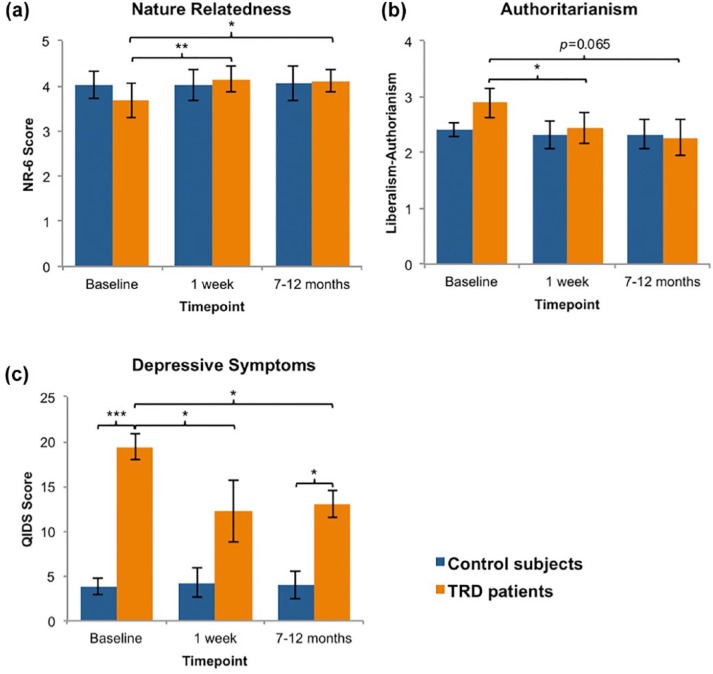
(a) Nature relatedness. Patients reported being significantly more connected to nature 1 week (*t*(6)=−4.242, *p*=0.003) and 7–12 months (*t*(5)=−2.707, *p*=0.021) after psilocybin treatment compared with baseline. No significant difference was found for the controls at the first (*t*(6)=0.008, *p*=0.994) or second follow-ups (*t*(5)=−1.228, *p*=0.274). (b) Political perspective. Patients were significantly less authoritarian 1 week after psilocybin treatment (*t*(6)=2.120, *p*=0.039) and a trend-level decrease was found at 7–12 months (*t*(5)=−1.811, *p*=0.065) compared with baseline. No significant differences were found for the controls at the first (*t*(6)=0.642, *p*=0.544) or second follow-up (*t*(5)=0.515, *p*=0.629). (c) Depressive symptoms. Patients had significantly more depressive symptoms than controls at baseline (*U*=0.0E0, *p*=0.001). One week after psilocybin treatment, depressive symptoms were significantly reduced to levels more comparable with controls (*Z*=−2.040, *p*=0.025) as no significant between-groups differences were found (*U*=10.000, *p*=0.062). The patients’ depressive symptoms remained significantly reduced at the 7–12-months follow-up (*Z*=−1.782, *p*=0.038); however, a between-groups difference was found (*U*=3.500, *p*=0.020). No significant differences were found for the control subjects at the first (*Z*=−0.422, *p*=0.673) or second (*Z*=−0.137, *p*=0.891) follow-ups compared with baseline. Data expressed as mean ± SEM [*p*<0.05*; *p*≤0.01**; *p*≤0.001***].

Patients showed a significant increase in nature relatedness scores 1 week after psilocybin treatment (*M*=4.14, *SD*=0.75) compared with baseline (*M*=3.67, *SD*=1.00); *t*(6)=−4.242, *p*=0.003, 95% CI [–0.75, 0.2]. The Hedges’ *g* value (*g*=2.5) for this contrast was very large, and the significant change was sustained at the 7–12-month follow-up time-point (*M*=4.12, *SD*=0.60; *t*(5)=−2.707, *p*=0.021, 95% CI [–1.15, –0.03]; *g*=1.2). Non-treated control subjects showed no difference between screening (*M*=4.02, *SD*=0.79) and the first (*M*=4.02, *SD*=0.94; *t*(6)=0.008, *p=*0.994, 95% CI [–0.46, 0.46]) or second (*M*=4.05, *SD*=0.62; *t*(5)=−1.228, *p*=0.274, 95% CI [–0.60, 0.21]) follow-ups. No significant between-groups differences were found at baseline (*p*=0.471) or the first (*p=*0.799) and second (*p*=0.861) follow-ups. These results suggest that psilocybin therapy increases the subjective sense of connectedness to nature 1 week after treatment, and that these effects are sustained for at least 7–12 months (Figure 1(a)).

### Political perspective

Patients showed a significant decrease in authoritarian political perspective 1 week after psilocybin treatment (*M*=2.43, *SD*=0.73) relative to baseline (*M*=2.89, *SD*=0.72); *t*(6)=2.120, *p*=0.039; 95% CI [–0.07, 0.99]. The Hedges’ *g* value (*g*=0.8) was found to meet the conventional criterion for a large effect size. Decreases in authoritarianism were still evident 7–12 months post-dosing (*M*=2.27, *SD*=0.68), and although this trend-level effect no longer reached conventions for statistical significance (*t*(5)=−1.811, *p*=0.065, 95% CI [–0.25, 1.46]), the relevant Hedges’ *g* value (*g*=0.7) met convention for a medium-to-large effect size, suggesting that this study was underpowered to detect a statistically significant result on this particular measure. Non-treated control subjects showed no change in authoritarianism between screening (*M*=2.40, *SD*=0.35) and the first (*M*=2.31, *SD*=0.66; *t*(6)=0.642, *p*=0.544) or second (*M*=2.33, *SD*=0.78; *t*(5)=0.515, *p*=0.629) follow-ups. No significant between-groups differences were found at baseline (*p*=0.132) or the first (*p=*0.755) or second (*p*=0.877) follow-ups. These results suggest that psilocybin therapy may persistently decrease authoritarian attitudes post-treatment with psilocybin, but that further research is required to test the robustness of this relationship (Figure 1(b)).

### Depressive symptoms

Baseline depressive symptoms (QIDS ratings) were significantly higher in patients (*M*=19.43, *SD*=4.04) than controls (*M*=3.86, *SD*=2.41); *U*=0.0E0, *p*=0.001 (Figure 1(c)). Further, the Hedges’ *g* effect size value for this contrast (*g*=4.7) was, by convention, exceptionally high, confirming our assumption that the patients had far higher (baseline) self-reported depressive symptoms than the controls.

Therapeutic outcomes for the full sample are given in previous publications (Carhart-Harris et al., 2016a, 2018a, 2017). In the sample reported in this paper, patients showed a significant decrease in QIDS scores 1 week after the psilocybin sessions (*M*=12.29, *SD*=9.18) compared with baseline (*M*=19.43, *SD*=4.04); *Z*=−2.04, *p*=0.025 (Figure 1(c)). Further, the Hedges’ *g* value for this contrast (*g*=1.3) was, again, large by convention. There was no significant difference found in the controls’ baseline (*M*=3.86, *SD*=2.41) versus 1-week follow-up QIDS scores (*M*=4.29, *SD*=4.35; *Z*=−0.422, *p*=0.673). These results confirm that depressive symptoms were significantly reduced for the patients 1 week after psilocybin treatment, but did not change over an equivalent period for the non-treated control subjects.

The patients’ 1 week post-treatment QIDS scores (*M*=12.29, *SD*=9.18) did not differ significantly from the controls’ follow-up values (*M*=4.29, *SD*=4.35; *U*=10.000, *p*=0.062), implying that the patients’ depressive symptoms were reduced to levels comparable with a healthy population at the 1-week post-treatment time-point (Figure 1(c)).

The patients’ QIDS scores remained significantly decreased 7–12 months after psilocybin treatment (*M*=13.00, *SD*=6.39) compared with baseline (*M*=19.43, *SD*=4.04); *Z*=−1.782, *p*=0.038. Moreover, the Hedges’ *g* value (*g*=0.7) for this contrast was ‘medium-to-large’. There was no significant difference found in the controls’ baseline (*M*=3.86, *SD*=2.41) versus 7–12-month follow-up QIDS scores (*M*=4.00, *SD*=3.58); *Z*=−0.137, *p*=0.891 (Figure 1(c)). These results confirm that the patients’ significant reductions in depressive symptoms were sustained for 7–12 months after psilocybin treatment, and did not change over an equivalent period for the non-treated control subjects.

## Discussion

The present study sought to investigate the effects of psilocybin with psychological support on nature relatedness and authoritarianism in patients with TRD. Patients reported a greater connection with nature 1 week after treatment. This increase in nature relatedness was sustained at the 7–12-months follow-up. One week post-treatment a significant decrease in authoritarianism was also observed, and at the 7–12-month follow-up the decrease was at trend level. No significant differences in nature relatedness, authoritarianism or depressive symptoms were found in an age and education-matched group of control subjects measured over an equivalent time period, thus supporting the inference that the changes were *not* due to order effects. Taken together, these findings indicate that the psychologically supportive administration of psilocybin might induce sustained changes in attitudes and beliefs, including feeling closer to nature and less allied to authoritarian views.

The reduction of depressive symptoms following psilocybin treatment found here is consistent with previous studies demonstrating the therapeutic potential of psychedelic compounds (Carhart-Harris and Goodwin, 2017). Long-term improvements in psychological wellbeing (Griffiths et al., 2008) and trait openness and optimism (Carhart-Harris et al., 2016b; MacLean et al., 2011; Schmid and Liechti, 2017) have been observed in healthy volunteers following a single exposure to a psychedelic drug. A single dose of psilocybin has also been shown to induce enduring reductions in anxiety and depression as well as increases in quality of life, life meaning, and optimism in patients with anxiety reactive to advanced-stage cancer (Griffiths et al., 2016; Grob et al., 2011; Ross et al., 2016). Furthermore, rapid and sustained antidepressant effects were found in patients with recurrent MDD that were treated with a single dose of the plant-based psychedelic brew, ayahuasca (Osorio Fde et al., 2015).

Evidence suggests that greater nature relatedness is associated with lower anxiety (Martyn and Brymer, 2014) and greater personal wellbeing (Capaldi et al., 2014; Cervinka et al., 2012; Howell et al., 2011; Mayer and Frantz, 2004; Mayer et al., 2008; Nisbet and Zelenski, 2011; Nisbet et al., 2011; Zelenski and Nisbet, 2014), and that exposure to awe-inspiring nature may increase pro-social behaviour (Piff et al., 2015) – perhaps through a related mechanism of seeing oneself as small in relation to the vastness of nature. Here, we show that the TRD patients felt more connected to nature up to 7–12 months after psilocybin treatment. This is consistent with a previous study in healthy participants in which 38% of the sample reported enduring positive changes in their relationship to nature and the environment 8–16 months post-psilocybin (Studerus et al., 2011). Psychedelic use in a large sample of web-survey respondents was found to positively predict nature relatedness (Nour et al., 2017); moreover, as with nature-inspiring experiences of awe (Piff et al., 2015), this relationship was strongest in people who experienced the greatest ego-dissolution during their most intense psychedelic experience (Nour et al., 2017).

Psychedelic users have been found to rate themselves as more concerned with the environment than users of other illicit substances (Lerner and Lyvers, 2006; Nour et al., 2017). Interacting with nature has been shown to have cognitive and affective benefits in healthy individuals (Berman et al., 2008) and patients with MDD (Berman et al., 2012). Although we found no correlation between the changes in nature relatedness or authoritarianism and changes in depression in the patients, this may simply have been due to the small sample size (*n*=7), or it is possible that a measure of mental wellbeing may have been more sensitive to such a relationship than the presently used measure of depression. There is, however, evidence to suggest that nature exposure decreases rumination as well as activity in brain regions implicated in depression (Hamilton et al., 2015), namely the subgenual prefrontal cortex (sgPFC) and regions of the default mode network (DMN) (Bratman et al., 2015). Interestingly, the administration of psilocybin has also been shown to acutely decrease sgPFC and DMN blood flow and within-network functional integrity (Carhart-Harris et al., 2012; Hamilton et al., 2015) and to increase global connectivity in the brain (Petri et al., 2014; Roseman et al., 2016). Further work is required to test the hypotheses that a renewed sense of ‘connectedness’, including feeling connected to nature, is a key factor determining therapeutic outcomes in psychedelic therapy (Carhart-Harris et al., 2018b; Watts et al., 2017b) and to better elucidate its basis in the brain. A working hypothesis is that increased global connectivity in the brain and its relationship to ego-dissolution (Tagliazucchi et al., 2016) and associated ‘connectedness’ (Watts et al., 2017b) is a key mediating factor (Carhart-Harris et al., 2018b).

Psychedelic drug use in the 1960s and 1970s was strongly associated with anti-establishment and egalitarian counter-culture movements (Nichols, 2016), yet very little controlled research has investigated the link between psychedelic use and political perspectives. Here we show for the first time, in a controlled study, lasting changes in political values after exposure to a psychedelic drug. This is in line with early research showing that recreational LSD users score higher on attitudes of ‘personal liberty’ and ‘foreign policy liberalism’ than control subjects (McGlothlin and Arnold, 1971). Psychedelic users have also been shown to score higher on ‘concern for others’ and place lower value on ‘financial prosperity’ than non-users of illicit substances as well as users of amphetamine, cannabis or heroin (Lerner and Lyvers, 2006). Given that psychedelics act through the serotonin system, it is interesting that serotonin has been implicated in the assessment of harm in moral decision-making, altruistic punishment and fairness (Crockett et al., 2008, 2010, 2013; Story et al., 2015). Moreover, psychedelics have been shown to increase trait openness (Carhart-Harris et al., 2016b; Griffiths et al., 2008; MacLean et al., 2011), and a substantial body of evidence demonstrates a positive association between openness and liberalism within individuals (Carney et al., 2008; Nour et al., 2017; Sibley et al., 2012; Xu et al., 2013). Relatedly, the role of the serotonin 2A receptor, the key site of action of psychedelics, in mediating ‘conversion-type’ or ‘quantum change’ (Miller, 2004) experiences has recently been discussed (Carhart-Harris and Nutt, 2017).

There are a number of important limitations to this study that must be considered when interpreting the results. The study formed part of an open-label clinical trial with a small sample size. The sample was smaller still for the NR-6 and PPQ-5, as these measures were introduced late in the trial due to inspiration from a separate project of ours (Nour et al., 2017). Also, although we recruited a control group to examine test–retest reliability on these measures, the controls were healthy subjects and were not exposed to the same treatment procedures. Critically, since treatment with psilocybin involved more than just drug administration (e.g. psychological support before and after the psilocybin dosing sessions), it is quite possible that drug-unrelated factors contributed to the changes in NR-6 and PPQ-5 scores observed here. The caring therapeutic model may have been one such factor. A large double-blind randomised control trial, ideally with an active control condition (to try and maintain the study blind), is required to more rigorously test the possible causal association between psilocybin and changes in nature relatedness and political perspective reported here. It would be hasty, therefore, to attempt any strong claims about a causal influence due specifically to psilocybin at this stage, and we should also be aware of anomalies in the relationship between psychedelic use and left-wing politics (Henrik, 2017); however, intriguing questions relating to psychedelics and political/philosophical perspectives remain.

A further limitation concerns the gender matching of control subjects and TRD patients; all TRD patients were male, whereas there were more females (71%) than males (29%) in the control condition. Thus, our findings in the TRD group cannot necessarily be extrapolated to females and the possibility of a gender effect cannot be discounted, and neither can we directly extrapolate the present findings to non-depressed populations.

The specificity of our main results also requires careful consideration. The question remains to be addressed whether the reported changes in nature relatedness and authoritarianism observed here post-treatment with psilocybin were selective for these outcomes, or rather an epiphenomenon of the treatment’s core effects on depressive symptoms. The question of causality is of central relevance here, and only further research can elucidate this. In this context, we would like to propose that there is a common mediating factor at play, driving both the improvements in mental health and changes in belief systems seen here – as well as elsewhere with psilocybin and other psychedelics (Carhart-Harris et al., 2016b; Hendricks et al., 2015b; Krebs and Johansen, 2013; MacLean et al., 2011). Such a common factor could be seen as a mental health equivalent of the general intelligence factor (e.g. Spearman’s *g*) in cognitive science (Spearman, 1987; Spiegelman, 2010). More specifically, in line with a recent commentary from our team (Carhart-Harris et al., 2018b) we propose that *connectedness* is this factor (see Carhart-Harris et al. (2018b); Watts et al. (2017b)), and that psychedelics positively and potently modulate this.

*Connectedness* is a construct in need of development, but related concepts can be found in the literature (Alexander, 2010). In brief, connectedness can be defined as a sense of feeling connected to one’s self (i.e. a sense of feeling emotionally and somatically integrated and at peace) as well as others (e.g. one’s partner, family, friends, colleagues and community) and the world more generally (e.g. feeling connected to nature and a guiding ethical and/or philosophical principle), as described in our recent work (Carhart-Harris et al., 2018b; Watts et al., 2017b). Thus, a remediating effect on feelings of disconnectedness, characteristic of a broad swathe of mental illness (Alexander, 2010), may underlie both the improvements in depressive symptoms and the relevant changes in political perspective reported here.

What of the association between mental health and political perspective? There is some support for a link between lower authoritarianism and better mental health (Amodio et al., 2007; Hirsh et al., 2010; Jost et al., 2003), although there are also some contradictory findings (Verhulst et al., 2016; Vigil, 2010). The idea that drugs, including legal ones such as alcohol (Nour et al., 2016) and caffeine (Carpenter, 2015), and medications such as stimulants (Ohler and Whiteside, 2017) and selective serotonin reuptake inhibitors (Kramer, 1997) can modulate belief systems, including political perspective, is relatively new – but one that may be fundamentally important, with potentially profound implications. If, for example, it was found that excessive alcohol use promotes a detachment from nature (Nour et al., 2016), chronic stimulant use promotes an aggressive industriousness and hubris – and potential for paranoia (Ohler and Whiteside, 2017) – and psychedelic experiences promote a generalised sense of connectedness (Carhart-Harris et al., 2018b; Watts et al., 2017b), including greater altruism (Forstmann and Sagioglou, 2017; Schmid and Liechti, 2017), what implications would this have for societies and their policies on such drugs? This and related topics may be seen as part of a new branch of political science, focused on the psychology and neurobiology of political perspective (Amodio et al., 2007; Everett et al., 2015; Haidt, 2013; Kanai et al., 2011; Kaplan et al., 2016; Nash et al., 2017).

Finally, we are keen to avoid a value judgement about the political changes that may (or may not) be attributable to psychedelic use. For some, ecological considerations (e.g. captured by nature relatedness) may be assigned the greatest importance (Lovelock and Hudson, 2016), particularly when considering the scale and seriousness of the problem posed by climate change, for example (Prüss-Ustün et al., 2016), whereas others may recognise ‘order’ (e.g. captured in part by our authoritarianism scale) as an essential and functional counterweight to lawlessness (Bejan, 2017; Haidt, 2013). Testing whether psychedelic use is ‘beneficial for society’ would be a complex project, not least because opinions will differ on how to define ‘beneficial’ (although see Harris (2010) for a thought-provoking discussion on this matter). Even so, exploring the potential of psychedelics to moderate extremist views, and/or facilitate reconciliation, might be worth exploring, given the present results and wider supporting literature (Amodio et al., 2007; Carhart-Harris et al., 2018b; Hirsh et al., 2010; Jost et al., 2003; Watts et al., 2017a).

In conclusion, this study sought to investigate the effects of psilocybin with psychological support on nature relatedness and authoritarian attitudes in patients with TRD. With significant caveats clearly highlighted, our findings tentatively raise the possibility that given in this way, psilocybin may produce sustained changes in outlook and political perspective, here in the direction of increased nature relatedness and decreased authoritarianism. These findings motivate further controlled studies to better determine the causality, reliability, specificity and durability of this relationship, as well as potential applications.

Before I enjoyed nature, now I feel part of it. Before I was looking at it as a thing, like TV or a painting… [But now I see] there’s no separation or distinction, you *are* it. *(Patient from this trial)*
